# The abstraction habituation model of knowledge worker burnout

**DOI:** 10.3389/fpsyg.2026.1763376

**Published:** 2026-01-21

**Authors:** James Meaden

**Affiliations:** Independent Researcher, Leesburg, VA, United States

**Keywords:** abstraction habituation, burnout, cognitive flexibility, environmental model of mindfulness, healing workplaces, knowledge work, mindfulness

## Abstract

Burnout among knowledge workers presents a puzzle: many workers report persistent burnout despite relatively favorable conditions, and interventions show limited lasting effects. Dominant resource models assume rest restores function, yet cannot fully explain why recovery fails despite adequate time, why burnout remains stable despite accumulating resources, or why mindfulness intervention effects fade when practice stops. Building on the environmental model of mindfulness, this paper introduces abstraction habituation: the progressive loss of cognitive flexibility through sustained knowledge work. The model proposes that neuroplastic adaptation establishes abstraction as the default processing mode, reducing the concrete processing capacity that supports psychological recovery. This framework accounts for career-long burnout stability and limited intervention durability through cumulative cognitive training, suggesting effective prevention requires redesigning work environments to preserve cognitive flexibility, not solely adding individual coping resources.

## 1 Introduction

Burnout among knowledge workers is pervasive. Global surveys consistently document high prevalence across knowledge-intensive sectors (e.g., Boston Consulting Group, 2024), and academic studies have identified high levels of burnout in knowledge-work roles ranging from software engineers (Tulili et al., 2023), to university professors (Fernández-Suárez et al., 2021), to finance professionals (Amigo et al., 2014). In line with this trend, the World Health Organization now recognizes burnout as an occupational phenomenon warranting thorough investigation (World Health Organization, 2019). Yet this prevalence presents a puzzle; not in the burnout rates themselves, but in the conditions under which burnout occurs in knowledge workers.

Knowledge workers often enjoy relatively favorable circumstances: flexible schedules, competitive compensation, intellectually stimulating work, and physical environments far removed from the industrial hazards that motivated early occupational health research. Yet they report high levels of burnout that current intervention approaches struggle to resolve. Common approaches including stress management programs (Awa et al., 2010), organizational changes such as workload reduction (Demerouti, 2024), and mindfulness training (Ong et al., 2024; Shoker et al., 2024) can provide relief from burnout symptoms, but these benefits often fail to persist beyond the intervention period. Even extended vacations, during which workers should theoretically replenish depleted resources, produce recovery that begins fading within days (de Bloom et al., 2009; Westman and Eden, 1997). The pattern across interventions is striking: they work temporarily, but something keeps reasserting itself (Awa et al., 2010; Demerouti, 2024). This suggests current approaches may be treating symptoms rather than causes, addressing surface manifestations while leaving underlying mechanisms intact.

The two dominant burnout frameworks, Maslach’s three-dimensional model (Maslach and Jackson, 1981) and Job Demands-Resources (JD-R) theory (Demerouti et al., 2001), share assumptions, as typically applied, that may limit their explanatory power for knowledge work contexts. For example, both (a) focus primarily on demand *quantity*—too many demands, insufficient resources, an imbalance requiring rebalancing, (b) employ an energy or resource metaphor—the depleted tank needs refilling, the overdrawn account needs deposits, and (c) implicitly assume that rest should restore function—that adequate time away from demands should return workers to baseline. Neither fully specifies why certain demands might produce qualitatively different kinds of depletion, nor why recovery capacity itself seems to erode over time rather than simply requiring more extensive restoration. While subsequent refinements have distinguished challenge from hindrance demands (Crawford et al., 2010), these categorize demands by motivational valence (i.e., whether they promote or thwart growth) rather than examining the cognitive character of the work itself. Therefore, a different kind of explanation may be needed, one focused on demand *quality* rather than quantity, on what *kind* of cognitive work is being demanded rather than how much.

Modern knowledge work demands sustained abstract processing, with many tasks requiring symbolic manipulation, conceptual analysis, future simulation, and strategic planning which are all conducted in representational space with minimal sensory grounding. This paper suggests that through neuroplastic adaptation, this sustained demand trains minds toward abstraction as the default processing mode, functioning as a kind of reverse mindfulness training that progressively weakens connection to present-moment sensory experience. Over time, the capability for concrete, present-moment, sensory-grounded processing is diminished—precisely the cognitive mode that supports psychological recovery (Kaplan, 1995; Sonnentag and Fritz, 2007; see Section 4). This is habituation to a particular cognitive style, which requires *retraining* to reverse; rather than resource depletion, which requires only rest. The abstraction habituation model presented in this paper attempts to explain why rest does not restore, why effects accumulate over career timescales, and why interventions that work temporarily fail to produce lasting change; they are competing against environmental training with insufficient countermeasures.

The abstraction habituation model builds on the Environmental Model of Mindfulness (EMM; Meaden, 2024a), which establishes that environments systematically shape whether minds default to concrete or abstract processing (see Section 2 for construct definitions). The EMM demonstrates that immediate-return environments, characterized by direct action-outcome feedback loops, sensory richness, and embodied engagement, naturally support concrete, present-moment awareness, what is commonly termed “mindfulness.” Delayed-return environments, lacking these characteristics, train minds toward abstract, future-oriented, self-referential processing. Modern knowledge work represents an extreme case of the delayed-return environment: maximally abstract, minimally embodied, chronically lacking immediate feedback. If environments can train minds toward concrete processing, as the EMM demonstrates, they might also train minds toward abstract processing as a default mode. The following sections develop this argument systematically, examining current burnout theory, the mindfulness-burnout relationship, the EMM, the abstraction habituation mechanism, its manifestation through specific work characteristics, its explanatory power for documented phenomena, and implications for theory, intervention, and research.

## 2 Key constructs

Before proceeding, it is helpful to define the central constructs precisely, as these terms carry specific meanings throughout this paper.

*Concrete processing* refers to the sensory-grounded, present-moment cognitive engagement with immediate environmental signals. It operates through low-level concrete predictions (Clark, 2013; Kiebel et al., 2008) tied to current perceptual input and is characterized by attention directed toward proximal, contextually-embedded experience. Phenomenologically, concrete processing corresponds to what mindfulness research describes as present-moment awareness.

*Abstract processing* refers to the conceptual, representational cognitive engagement decoupled from immediate sensory input. It operates through high-level abstract predictions (Clark, 2013; Kiebel et al., 2008) that transcend current perceptual context, manipulating representations across temporal, spatial, and hypothetical dimensions. Phenomenologically, abstract processing encompasses planning, rumination, self-evaluation, and mental simulation—cognitive activities that mindfulness practices aim to quiet.

*Cognitive flexibility*, as used here, denotes the capacity to shift fluidly between abstract and concrete processing modes as situational demands require. This is distinct from the broader construct of cognitive flexibility in the neuropsychological literature (e.g., set-shifting; Miyake et al., 2000); the usage in this paper refers specifically to flexibility along the abstract-concrete dimension of cognitive processing.

*Abstraction habituation* is the progressive reduction in cognitive flexibility—specifically, the diminished capacity to downshift from abstract to concrete processing—that develops through sustained environmental training toward abstraction. It represents not a skill deficit but an adaptive specialization: the brain optimizing for what it repeatedly does. The term “habituation” emphasizes that this is a trained pattern.

Concrete and abstract processing are not proposed as discrete categories but as poles of a continuum. Healthy cognition involves dynamic movement along this continuum; abstraction habituation represents this movement becoming constrained toward one pole.

## 3 Current state of burnout theory

Christina Maslach’s foundational work established both the conceptual and measurement architecture for burnout research (Maslach and Jackson, 1981). The three-dimensional model identifies emotional exhaustion, depersonalization or cynicism, and reduced personal accomplishment as the core components, with exhaustion recognized across virtually all subsequent definitions as the central element of burnout (Maslach et al., 2001; Shirom, 2003).

The Maslach Burnout Inventory (MBI; Maslach and Jackson, 1981) remains the gold-standard measurement tool, originally developed in human services contexts and subsequently adapted for broader occupational application (Schaufeli et al., 1996). Maslach and Leiter’s Areas of Worklife Model extended this foundation by identifying six domains of person-job mismatch (i.e., workload, control, reward, community, fairness, and values) predicting burnout risk (Leiter and Maslach, 2004). This work defined what burnout is, but did not yet specify the mechanism by which mismatch in these domains produces burnout.

Job Demands-Resources (JD-R) theory (Bakker and Demerouti, 2017; Demerouti et al., 2001) provides the dominant explanatory framework for burnout, proposing that demands deplete energy while resources buffer demands and promote engagement. The theory identifies two pathways: health impairment, through which demands lead to exhaustion, and motivation, through which resources lead to engagement. Conservation of Resources (COR) theory (Hobfoll, 1989) complements this framework by emphasizing resource loss spirals, in which initial resource loss makes further loss more likely, and the greater psychological impact of losses relative to equivalent gains. These theories have substantial empirical support and considerable flexibility in application across contexts (i.e., both provide broad and relatively flexible categories for different types of resources and demands). Their limitation for present purposes lies in the energy metaphor previously mentioned and frequently used by these frameworks: if burnout results from depleted resources, rest and resource acquisition should restore function. The persistence of burnout despite adequate recovery time (de Bloom et al., 2009; Westman and Eden, 1997), the stability of burnout at elevated levels despite accumulating resources and autonomy across careers (Dunford et al., 2012; Mehta et al., 2019), and the persistent cognitive and health consequences of early-career burnout episodes years after the initial episode (Rudman et al., 2020) all suggest mechanisms beyond just resource dynamics. The model developed in this paper proposes abstraction habituation as one such mechanism. Importantly, the purpose of the model presented in this paper is not to replace resource models but to complement them by addressing phenomena they struggle to explain.

Recent burnout research increasingly recognizes cognitive impairment as central rather than peripheral to the syndrome (Deligkaris et al., 2014; Kulikowski, 2021). Demerouti and colleagues note the growing recognition of “cognitive weariness” as a potential core dimension (Demerouti et al., 2021), with some researchers now proposing four-component models that include cognitive impairment alongside exhaustion, mental distance, and emotional impairment (Schaufeli et al., 2020). Documented cognitive deficits associated with burnout include impaired executive function, working memory, attention, and decision-making (Deligkaris et al., 2014; Jonsdottir et al., 2013). Research by Beck et al. (2013) demonstrates that executive function performance can recover to healthy control levels following intervention, suggesting these impairments can represent state-dependent dysfunction rather than permanent decline. However, recovery trajectories vary considerably; while some individuals show improvement within 12 weeks with targeted interventions such as aerobic exercise (Beck et al., 2013), others demonstrate only partial cognitive recovery even after 1.5 years (Oosterholt et al., 2016), and longitudinal research indicates that cognitive complaints can persist for several years following clinical improvement (Dalgaard et al., 2021; Ellbin et al., 2018). Notably, this entire body of research shares a common assumption: that cognitive impairment results *from* burnout. The possibility that certain cognitive processes might instead contribute *to* burnout—functioning as mechanism rather than symptom—remains largely unexplored.

Despite decades of research, fundamental questions about burnout mechanisms remain unresolved. As Mańkowska (2025) observes, the field still lacks theoretical integration, with competing paradigms dividing rather than unifying scientific communities. This lack of theoretical integration has left key questions inadequately explained: why burnout stabilizes at elevated levels rather than progressively worsening or recovering, and why recovery capacity erodes rather than simply requiring restoration. Addressing these gaps may require looking beyond the demand-resource frameworks that have dominated the field. One literature that has received substantial attention yet remains undertheorized is the relationship between mindfulness and burnout, a relationship that, properly reframed, may help to illuminate the cognitive mechanisms underlying the syndrome.

## 4 Mindfulness and burnout

Evidence demonstrates that mindfulness-based interventions reduce burnout symptoms (Ruotsalainen et al., 2015; Shoker et al., 2024; Wang et al., 2023). Meta-analyses consistently support beneficial effects on emotional exhaustion in particular (Shoker et al., 2024; Wang et al., 2023), with benefits observed across industries and occupational groups. Mindfulness has become the predominant individual-level intervention in burnout research, with one recent systematic review identifying 49 randomized controlled trials (RCT) examining standardized mindfulness programs for burnout over a ten-year period (Shoker et al., 2024). These interventions are typically positioned as evidence-based approaches for helping workers manage occupational stress. Proposed mechanisms include increased emotional regulation capacity, reduced rumination, enhanced attentional control, and improved coping resources (Irving et al., 2009; Shapiro et al., 2006). Within the dominant theoretical frameworks, mindfulness functions as a personal resource that can be acquired through training, and that buffers the impact of job demands on burnout.

This resource-addition framing carries implicit assumptions worth examining. The intervention logic runs: (1) workers experience burnout because they lack adequate coping resources; (2) mindfulness training adds a resource (present-moment awareness, non-reactive attention); (3) this additional resource helps workers manage demands that would otherwise overwhelm them; (4) burnout decreases as a result. The model places explanatory weight on individual capacity; workers who burn out lack something, and training provides what’s missing. Responsibility for burnout prevention shifts substantially to the individual, who must acquire and maintain the skill. The work environment remains as it was, the worker simply becomes better equipped to handle it. Yet this framing leaves something underexplained. If mindfulness adds a skill or resource, one might expect that capacity to persist after training ends, as with other trained skills (Arthur et al., 1998). Occasional practice may be required to prevent skill decay, but not continuous engagement merely to maintain baseline. Yet follow-up data suggest these benefits may attenuate over time, with researchers noting that mindfulness likely requires ongoing practice for long-term maintenance (Shoker et al., 2024). This phenomenon suggests that mindfulness may not be adding something new, so much as temporarily counteracting something ongoing. This paper poses the following questions: What if the causal arrow runs in the other direction? What if something is being continuously eroded, and mindfulness practice temporarily restores it?

To identify what might be being eroded, consider what mindfulness training actually entails. The core instruction across mindfulness traditions, to attend to present-moment sensory experience without mental elaboration, is fundamentally an instruction to shift from abstract to concrete processing. When practitioners notice the breath, observe bodily sensations, or attend to sounds without labeling, they engage precisely the sensory-grounded, present-focused cognitive mode that abstract work systematically neglects. From this perspective, the effectiveness of mindfulness training on burnout symptoms may be less about adding a new personal resource and more about exercising a processing mode that knowledge work causes to atrophy. The claim here is not merely that this mode goes unused; it is that sustained disuse, combined with the continuous training of abstract processing, actively degrades the capacity for concrete processing. What is diminished is the very thing mindfulness training cultivates.

This interpretation finds partial support in established recovery research, though it extends beyond existing frameworks. Attention Restoration Theory (Kaplan, 1995) proposes that directed attention—the effortful, top-down processing required for planning, problem-solving, and sustained focus—fatigues with use and requires a qualitatively different mode to restore. Natural environments promote recovery by engaging “soft fascination,” an involuntary, bottom-up attention drawn by sensory interest rather than maintained by cognitive effort. The implication is that recovery requires actually shifting modes; continued abstract processing, even on different content, prevents restoration. Sonnentag’s research on psychological detachment (Sonnentag and Fritz, 2007; Sonnentag et al., 2010) converges from a different direction: workers who mentally disengage from work during off-hours show better recovery than those who continue ruminating or problem-solving. The key variable is cognitive mode, not rest per se. Present-moment sensory engagement—through activities such as nature exposure, mindfulness practice, or any activity that anchors attention in immediate sensory experience rather than abstract representation^1^—accomplishes the necessary shift.

These frameworks explain why concrete processing aids recovery, but they stop short of the present claim. Kaplan’s model treats concrete processing as instrumentally valuable, a way to rest directed attention so it can return to productive work. The present argument goes further: concrete, sensory-grounded processing may be intrinsically necessary for well-being, not merely a recovery tool for restoring abstract processing capacity. Sustained deprivation of concrete engagement, not just fatigue from overusing abstract processing, may itself constitute a form of impairment. If so, the short-lived benefits of mindfulness training on burnout symptoms might reflect not skill decay but the continuous pressure of environments that demand abstract processing and provide insufficient engagement of concrete processing networks.

This generates a different causal story than the dominant framing, and the difference matters for both theory and intervention. The traditional model positions mindfulness as additive: demands deplete resources, mindfulness training adds a coping resource, burnout decreases. The alternative presented here positions mindfulness as restorative: certain environments progressively erode the capacity for concrete processing, this erosion impairs both recovery and well-being directly, burnout develops, and mindfulness practice temporarily restores what the environment continuously degrades. Same phenomenon, opposite causal direction, and substantially different intervention implications. The traditional framing suggests helping individuals cope with demanding environments; the alternative suggests examining what about those environments diminishes capacity for concrete processing in the first place. If this reframe is correct, interventions targeting individuals alone face an uphill battle against continuous environmental pressure, and the priority shifts from building individual resilience to redesigning the environments that erode it. The EMM (Meaden, 2024a) provides theoretical architecture for this alternative framing.

## 5 The environmental model of mindfulness

The EMM reconceptualizes mindfulness as environmentally trained cognitive styles rather than solely an individual practice or trait (Meaden, 2024a). Where traditional approaches treat mindfulness as a skill cultivated through deliberate practice or a disposition varying across individuals (Baer et al., 2006; Brown and Ryan, 2003), the EMM proposes that environments systematically shape whether minds default to concrete, present-moment processing or to abstract, future-oriented, self-referential processing. As previously mentioned (see Section 2), phenomenological characteristics of mindfulness—present-moment awareness, non-judgmental attention to current experience, reduced identification with mental contents—correspond to what cognitive science describes as concrete processing: direct engagement with immediate sensory reality rather than abstracted representations of it (Laukkonen and Slagter, 2021). Conversely, the mental states that mindfulness practices aim to quiet—mind-wandering, future scenario simulation, rumination, worry, self-referential evaluation—correspond to abstract processing: mental operations that transcend immediate experience to manipulate representations across time, space, and hypothetical possibility (Laukkonen and Slagter, 2021). The EMM thus integrates mindfulness with established cognitive science frameworks, grounding these experiential phenomena in the abstract-concrete cognitive processing continuum (Clark, 2013; Friston, 2010; Kozhevnikov et al., 2014; Trope and Liberman, 2010).

### 5.1 Core framework

One particularly powerful example from the EMM is the distinction between immediate-return and delayed-return socioecological systems. Immediate-return societies, exemplified by hunter-gatherer groups, display direct relationships between action and outcome: effort expended today yields results today (Brunton, 1989; Martin, 1999; Martin and Shirk, 2008; Woodburn, 1982). Delayed-return systems, characteristic of modern economies, involve extended temporal gaps between effort and result, imposing cognitive demands for sustained abstract planning and future orientation (Martin, 1999; Woodburn, 1982). These represent not value judgments about social organization but descriptions of different cognitive demands. Immediate-return environments are characterized by concrete tasks with visible feedback, embedded social context, and immersion in natural, sensory-rich environments. Delayed-return environments involve abstract planning for future outcomes, ambiguous success criteria, infrequent feedback, and constructed environments that filter sensory experience. As mentioned previously, modern knowledge work represents an extreme case of a delayed-return environment: defined by symbolic manipulation and abstract representation (Pyöriä, 2005), it lacks the immediate feedback that characterizes immediate-return conditions. The evolutionary context here matters: human cognition developed over approximately 300,000 years of *Homo sapiens* existence (Hublin et al., 2017), with more than 95% of that time spent in immediate-return environments (Martin, 1999; Woodburn, 1982). The cognitive architecture we inherit, including the neural systems supporting concrete present-moment processing, was shaped by and for these conditions. Delayed-return environments, and the abstract processing they demand, represent an evolutionary eyeblink.

The EMM identifies four environmental factors that train concrete, present-moment processing: (1) *immediate feedback* provides clear action-outcome loops where efforts produce visible results, grounding cognition in sensory confirmation rather than abstract prediction (Martin, 1999; Martin and Shirk, 2008; Martin et al., 2014), while feedback delays impair sustained attention and reduce flow states (Nakamura and Csikszentmihalyi, 2002; Roche and McConkey, 1990; Sipowicz et al., 1962); (2) *experiential and observational learning* involves acquiring knowledge through direct engagement and watching others rather than through symbolic instruction, maintaining connection to concrete processing (Gaskins and Paradise, 2010; Paradise and Rogoff, 2009); (3) *nature exposure* immerses individuals in sensory-rich environments that reduce cognitive clutter and support sustained present-moment attention (de Fockert et al., 2011; Snell and Simmonds, 2012; Tang et al., 2022); (4) *interconnected self-construal* broadens identity to include the natural and social environment while reducing identification with internal mental states, thereby diminishing the self-referential abstract processing characteristic of independent self-construal (Lillard, 1998; Markus and Kitayama, 1991; Meaden, 2024a).

The crucial insight of the EMM is that mindfulness is not necessarily an advanced attentional skill to cultivate through training, but a natural cognitive baseline; what minds do when environmental conditions support, or even require, it. Evidence from immediate-return societies (Everett, 2010; Myers, 1991; Turnbull, 1983; Woodburn, 1982) and from environmental psychology research on nature exposure (Barbaro and Pickett, 2016; Caparos et al., 2012; de Fockert et al., 2011) converges on this point: concrete, present-focused awareness represents a natural mode that environments can either support or undermine. Therefore, modern minds are not inherently distracted or deficient in attention; many have been trained toward abstraction by environments that demand and reward abstract processing. The default mode network (DMN) activity observed during rest in subjects living in modern delayed-return environments (Henrich et al., 2010)—characterized by self-referential thought, future simulation, and mind-wandering (Killingsworth and Gilbert, 2010; Margulies et al., 2016; Menon, 2023)—may not represent a biological default but an environmentally trained cognitive baseline. Meditation research demonstrating that experienced practitioners show reduced DMN activity and a more present-centered default mode (Brewer et al., 2011) supports this interpretation. If cognitive baselines can be trained in one direction, then might they also be trained in the other? Either through intentional practice or environmental conditioning.

### 5.2 Theoretical alignment

The EMM aligns mindfulness with established cognitive science frameworks. Predictive processing theory (Clark, 2013; Friston, 2010) describes cognition as hierarchically organized prediction, with concrete sensory-specific predictions at lower levels and abstract context-invariant predictions at higher levels. Construal level theory (CLT) documents that psychological distance—temporal, spatial, social, or hypothetical—systematically shifts processing toward abstraction, with distant objects represented in high-level, decontextualized terms and proximal objects in concrete, contextualized detail (Trope and Liberman, 2010). Kozhevnikov et al.'s (2014) comprehensive taxonomy of cognitive styles similarly organizes cognition along a concrete-abstract axis, characterizing styles as adaptive patterns shaped by environmental demands. Emerging approaches in 4E cognition (embodied, embedded, enacted, extended) emphasize that cognition extends beyond the skull into bodily and environmental context (Newen et al., 2018), with the abstract-concrete dimension reflecting the degree of embodied grounding. These frameworks converge on the abstract-concrete dimension as fundamental to understanding how minds work and how environments shape mental functions.

### 5.3 Evidence for environmental training

Additional evidence supports environmental training of cognitive processing styles at population levels. James Flynn’s research on generational cognitive change (i.e., the Flynn Effect) documents substantial increases in abstract reasoning capacity across the twentieth century, gains too rapid to reflect genetic change (Flynn, 2007, 2012). Flynn interprets this as environmental training: exposure to formal education, urbanization, media, and modern work trained populations toward abstract classification and hypothetical reasoning. As Flynn (2012) describes it, modern minds have donned “scientific spectacles,” now approaching reality through conceptual frameworks rather than direct sensory experience. Luria’s pioneering Soviet expeditions of the 1930s demonstrated this environmental training effect by comparing groups with varying exposure to modern institutions. Studying illiterate peasants in remote Central Asian villages, Luria (1976) found that subjects organized cognition around concrete, situational relationships rather than abstract categories. When presented with hammer, saw, log, and hatchet, villagers grouped items by practical function and use-context rather than taxonomic category, refusing to exclude the log as a non-tool because it belonged with the implements that would act upon it. When asked syllogistic questions about bears in regions they had never visited, subjects declined to reason from premises alone, insisting that knowledge required direct experience rather than logical inference. This was not cognitive deficit but a fundamentally different processing style optimized for concrete environmental navigation. Critically, cognitive adaptations to abstract processing occurred rapidly with environmental change: illiterate peasants continued to use situational rather than categorical classification, while individuals with even brief schooling shifted dramatically toward abstract categorization (Luria, 1976). Contemporary research provides further cross-cultural evidence of this effect. Thong et al. (2024) documented cognitive style shifts among the Temiar people of Malaysia, an Indigenous group whose traditional rainforest subsistence lifestyle supported concrete, externally-focused cognition. Over the past two decades, some Temiar converted to Christianity and adopted more individualistic practices, producing systematic cognitive transformation. As Thong et al. summarize: “In sum, conversion impacts on self-construal (from collective to individual), perceptions (perceptual-concrete to conceptual-abstract), and emotional processing (from experience-near to experience-distant [more intellectualized emotional processing]; from external to internal focus) of the converts” (p. 8). The Temiar case demonstrates that immersion in environments with increasing demands for abstract processing trains minds toward abstraction on career-relevant timescales; precisely the mechanism proposed here for knowledge work.

### 5.4 From population to individual timescales

The evidence reviewed above strongly suggests that abstract and concrete processing modes represent adaptive responses to different environmental demands, and these environmental demands train cognitive defaults at population scales over generational timescales. The abstraction habituation model of burnout proposed in this paper extends this principle to individual scales over career timescales: if decades of modernization train populations toward abstraction, years of knowledge work may train individual minds in the same direction. The abstraction habituation model predicts that sustained immersion in such conditions progressively trains minds toward abstract processing while weakening the concrete processing capacity that supports burnout recovery and prevention.

## 6 Abstraction habituation: the core mechanism

### 6.1 Definition

As described earlier, abstraction habituation is the progressive loss of cognitive flexibility—specifically, the diminished capacity to shift from abstract conceptual processing to concrete sensory-grounded awareness—that can develop through sustained exposure to knowledge work environments demanding a high degree of abstract processing. Critically, abstraction habituation is proposed as a causal mechanism contributing to burnout, not a component or dimension of burnout itself. Burnout remains defined as the experiential syndrome characterized by exhaustion, cynicism, and reduced efficacy (Maslach and Jackson, 1981); abstraction habituation represents one underlying cognitive pathway through which certain work environments produce this syndrome.

### 6.2 Neuroplastic mechanism

The neuroplastic basis for abstraction habituation rests on established principles of use-dependent plasticity. Neural circuits that are repeatedly activated undergo structural and functional strengthening, while circuits receiving insufficient activation weaken over time (Draganski et al., 2004; Pascual-Leone et al., 2005). These principles operate across domains including motor learning, language acquisition, and expertise development (e.g., Woollett and Maguire, 2011).

Applied to the abstract-concrete processing dimension, sustained knowledge work provides intensive daily training of abstract processing networks while sensory processing networks, interoceptive circuits, and other systems supporting concrete present-moment awareness receive comparatively little activation during work hours. Over career timescales, this differential activation should produce shifts in the relative strength and accessibility of these processing modes—not damage or loss, but recalibration of defaults. The essence of the model is that the brain optimizes for what it repeatedly does: abstract processing networks strengthen and become increasingly automatic, while concrete sensory processing networks weaken from disuse. Importantly, this is adaptation, not pathology. The problem is that optimization for abstract work performance might simultaneously impair the cognitive flexibility necessary for psychological recovery.

Evidence for such training effects exists. Meditation research demonstrates that sustained practice produces structural changes in brain regions associated with attention and interoception (Fox et al., 2014; Hölzel et al., 2011), confirming that concrete processing networks can be strengthened through deliberate training. The population-level shifts toward abstract reasoning documented by Flynn (2007, 2012) indicate that abstract processing can similarly be shaped by environmental exposure. Abstraction habituation extends this logic to individual timescales: if environments train cognitive defaults at population scales over generations, the same mechanisms should operate at individual scales through career-length immersion in cognitively specialized work.

### 6.3 Cognitive manifestation

Flynn (2007) concept of “scientific spectacles” provides a useful metaphor for understanding what abstraction habituation does to cognitive flexibility. Pre-modern cognition operated through what might be called utilitarian spectacles—direct sensory experience, concrete practical knowledge, contextual understanding. Modern cognition operates through Flynn’s scientific spectacles—abstract classification schemes, conceptual frameworks, taxonomic organization (Flynn, 2007, 2012). We classify animals by biological category rather than by what they do in our experience; we understand causation through abstract mechanisms rather than concrete sequences. This shift represents trained capacity, not only biological evolution; they are the spectacles we have learned to wear. However, healthy cognition likely involves flexible access to both modes: workers can put on scientific spectacles when abstract thinking serves, and remove them when concrete engagement would serve better. Abstraction habituation represents this flexibility made rigid. Some knowledge workers may progressively lose the capacity to remove the abstract lens. Even outside work, the mind continues planning, simulating, analyzing, and worrying—about work-related matters and beyond—because abstraction has become its trained default processing mode. The experience is familiar to most knowledge workers: lying on a beach or hiking a trail, surrounded by sensory richness, yet unable to stop mentally rehearsing next week’s presentation or analyzing a colleague’s email.

### 6.4 The abstraction habituation cycle

It is proposed that, once established, abstraction habituation creates a self-reinforcing cycle. This cycle operates through four interconnected mechanisms. First, repeated abstract work strengthens abstract networks while concrete networks weaken from disuse, making abstraction increasingly automatic and default. Second, this trained default continues operating outside work—rumination about problems, mental rehearsal of meetings, planning and evaluation—preventing the shift to concrete sensory-grounded states that facilitate recovery. Third, without recovery, burnout accumulates. Fourth, burnout and its associated exhaustion impairs cognitive control—the effortful capacity to override automatic processing—which means greater reliance on default processing, which has been trained to be abstract. The cycle accelerates: more abstraction produces less recovery, more burnout, less cognitive control, and greater reliance on automatic abstraction (Figure 1).

**Figure 1:**
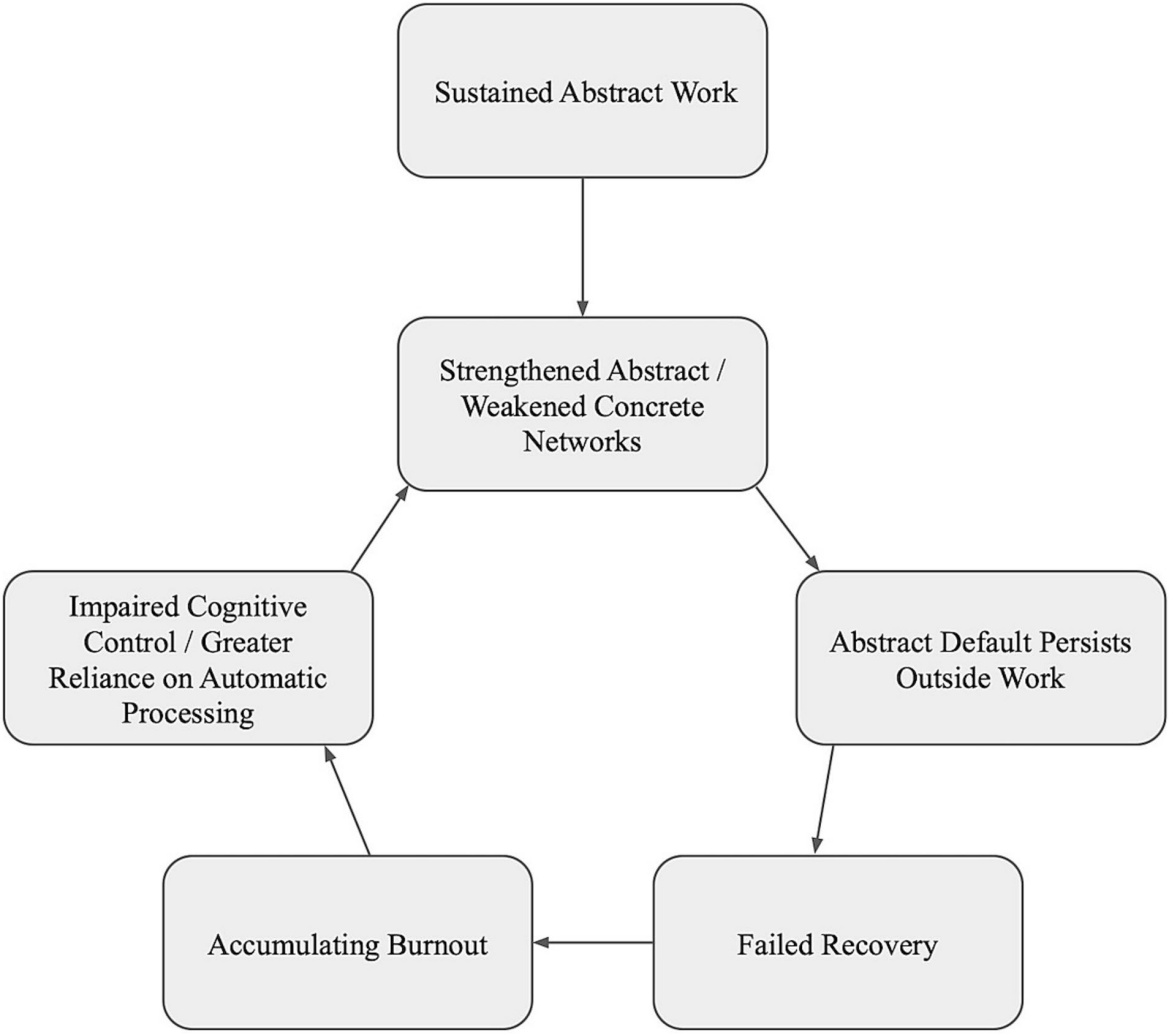
The abstraction habituation cycle. Sustained abstract work strengthens abstract processing networks, while concrete processing capacity weakens from disuse. Failed recovery leads to burnout, which impairs cognitive control and increases reliance on automatic processing, which has been trained toward abstraction.

### 6.5 Career trajectory implications

This self-reinforcing dynamic suggests that knowledge work career tenure predicts habituation severity independent of current workload because the training accumulates over time. Early-career workers retain flexibility from more cognitively diverse pre-work experiences. Mid-career workers, having fully adapted to decades of full-time abstract processing demands, show peak habituation levels. Late-career patterns likely reflect selection effects: workers with severe habituation exit the profession, while those remaining represent either greater baseline resilience or more favorable environmental fit.

## 7 Three work characteristics driving abstraction habituation

This section proposes three characteristics of modern knowledge work that create the training environment that produces abstraction habituation over career timescales. Each characteristic operates through a distinct mechanism: *abstraction load* concerns the type of processing demanded, *feedback deprivation* concerns the absence of immediate concrete performance signals, and *closure deprivation* concerns the absence of natural task endpoints.

### 7.1 Abstraction load

Abstraction load is the cognitive demand arising from sustained engagement with disembodied, symbolic, conceptually-mediated work lacking in sensory or environmental grounding. This is distinct from cognitive load generally, as it is specifically the demands of tasks requiring a high level of abstract processing. Work with a high abstraction load involves tasks that exist only or mostly in conceptual space, with minimal tangible referents to manipulate and limited sensory confirmation of progress. Examples of tasks with high abstraction load in modern knowledge work include: strategic planning without the direct observation of concrete outcomes, financial modeling without handling physical resources, policy development without witnessing effects on actual people, report writing about activities never directly observed, and project management through abstract dashboards rather than direct observation of the actual work being managed.

High abstraction load contributes to abstraction habituation because successful work performance requires sustained abstract processing. Completing the large-scale project, receiving a positive annual performance review, and then getting the promotion (and the next, and the next) all depend on maintaining a high degree of effective abstract processing over long periods of time. In this context, cognitive functioning optimizes for what produces these rewards, and abstract processing becomes increasingly automatic, efficient, and default. Simultaneously, concrete processing networks receive little activation during work hours and weaken from disuse. The cognitive flexibility to downshift—to move from conceptual analysis to sensory presence—progressively erodes. Paradoxically, workers who become most expert at their abstract work may become least capable of the concrete presence that would restore them from it. This paradox finds support in Kulikowski (2021) proposition that cognitive ability functions as both resource and demand; the capacity enabling performance can also generate strain. Abstraction habituation extends this insight: the adaptation enabling expert abstract work can simultaneously erode recovery capacity.

### 7.2 Feedback deprivation

Feedback deprivation is the absence of clear, immediate, unambiguous signals on work quality and progress, forcing an increased reliance on internal evaluation and abstract prediction rather than concrete environmental feedback. Knowledge work characteristically involves elongated action-outcome loops where weeks, months, or even years can separate effort from result, subjective and often ambiguous success criteria where it remains unclear whether work is “good enough,” dependence on others’ subjective evaluations rather than tangible measures of performance, and projects that are never truly complete but remain perpetually revisable. Clear signals of success (e.g., table successfully built, garden successfully planted) are often absent or delayed.

This feedback deprivation intensifies abstract processing by eliminating the frequent concrete feedback that would otherwise anchor cognition to the present. Without reliable environmental signals the mind generates predictions internally—producing the self-referential evaluation loops experienced as rumination about work quality, competence, and worth. Intrinsic reward systems that evolved for concrete task completion and the satisfaction of seeing finished results receive insufficient activation. Efficacy often becomes a matter of abstract self-assessment rather than clear environmental feedback. This means that the mind must engage continued abstract processing to not only produce but also *evaluate* abstract work outputs, creating a loop that reinforces abstract processing as the default mode. Research on role ambiguity demonstrates this pattern empirically: uncertainty about expectations and feedback increases abstract self-referential thought and reduces concrete present-moment awareness (Meaden, 2024b).

### 7.3 Closure deprivation

Closure deprivation is the absence of natural endpoints in abstract work, the structural lack of moments when tasks are definitively complete and can be mentally released. Unlike concrete tasks that reach physical completion (e.g., the field is plowed, the wall is built), abstract work often lacks natural closure. Work exists in conceptual space, accessible anywhere via thought; unresolved problems remain “open” mentally because no physical state marks their resolution. Digital accessibility extends work’s presence (e.g., email and messaging platforms maintain work in ambient awareness).

Therefore, closure deprivation extends abstract processing into would-be recovery time, compounding the training effect. Recovery from abstraction habituation requires shifting to concrete, sensory-grounded states which engage different neural networks and interrupt abstract processing patterns. But without closure, abstract work follows workers home mentally, converting what should be opportunities for concrete recovery time into additional abstract processing time. Even workers who maintain good work-life boundaries in terms of formal hours may find their minds continuing to process work abstractly. Research on psychological detachment demonstrates that work-related cognition routinely continues beyond formal work hours. Sonnentag (2012) found that the inability to mentally disconnect from work during non-work time is one of the strongest predictors of failed recovery, with workers who continue thinking about work problems showing elevated strain and reduced life satisfaction regardless of actual workload. This creates conditions where habituation intensifies even if formal workload decreases; the training continues during “recovery” time that is not actually providing full recovery because the processing mode has not sufficiently changed.

These three characteristics (high abstraction load, high feedback deprivation, high closure deprivation) are proposed as core environmental demands that lead to abstraction habituation. To summarize: high abstraction load trains abstract processing as the default mode, feedback deprivation maintains internal abstract evaluation as the main performance confirmation mechanism, and closure deprivation extends abstract processing into non-work time (Figure 2).

**Figure 2:**
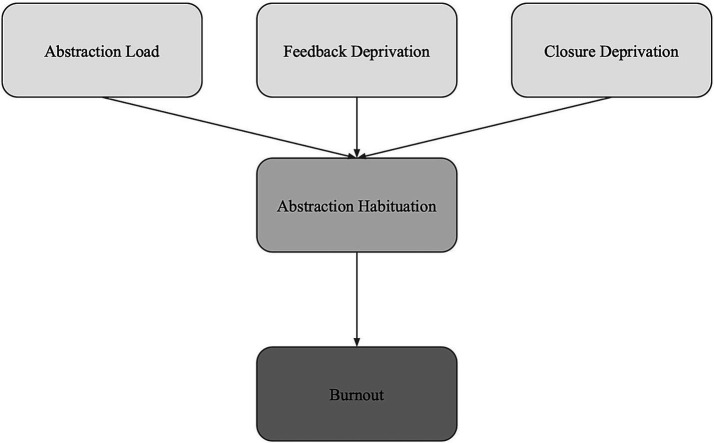
Three work demands driving abstraction habituation. Abstraction load (type of processing demanded), feedback deprivation (absence of objective performance signals), and closure deprivation (absence of natural endpoints).

## 8 Explanatory power

Abstraction habituation explains several well-documented phenomena that current burnout frameworks struggle to fully account for.

### 8.1 Perseverative cognition

Perseverative cognition—the continued cognitive representation of stressors even when the stressors are absent—is well-established as a predictor of poor recovery and adverse health outcomes (Brosschot et al., 2006; Ottaviani et al., 2016), including burnout (McCarrick et al., 2023). The phenomenon includes rumination, worry, and anticipatory stress, and it prolongs the physiological stress response beyond the actual presence of stressors (Verkuil et al., 2010; Ottaviani et al., 2016). Current explanations point to individual tendencies, stress response mechanisms, or negative affectivity, but these accounts do not explain *why* the mind keeps engaging in this processing. What mechanism sustains cognitive representation of stressors when the stressors themselves are not present?

Abstraction habituation explains perseverative cognition as the natural output of a mind trained to default to abstract, representational processing. The mind keeps ruminating because abstract processing is its trained mode. The *content* of perseverative cognition concerns stressors, but the *mode*—abstract representation, mental simulation, evaluative processing—is the habituated pattern itself. From this perspective, perseverative cognition is not an aberrant stress response, but rather it is what abstraction-habituated minds do as their default. The question shifts from “why do stressed workers ruminate?” to “why would minds trained for sustained abstraction not ruminate?”

### 8.2 Failed recovery

Knowledge workers frequently experience insufficient recovery. Vacation effects are particularly striking: workers report genuine recovery during extended time away, yet this recovery typically evaporates within weeks of returning to work. Westman and Eden (1997) found that burnout declined during vacation but returned completely to pre-vacation levels within three weeks, and Kühnel and Sonnentag (2011) found similar fade-out within one month. Current explanations involve insufficient detachment, continued demands through digital connectivity, or inadequate duration. But these explanations do not fully account for the persistence of the pattern: why the underlying condition reasserts itself so rapidly regardless of the quality or duration of the break.

Abstraction habituation explains recovery failure as the expected result of a trained cognitive pattern that travels with the person. Rest does not undo habits. Physical location changes during vacation, but the trained processing mode does not. On vacation, the abstraction-habituated mind continues planning, evaluating, simulating, and worrying. Therefore, vacation is insufficient to retrain patterns established over years of daily reinforcement. Effects fade upon return because the abstracting environment immediately re-engages these trained patterns.

### 8.3 Mindfulness effects that fade

Mindfulness interventions consistently reduce burnout symptoms (Bartlett et al., 2019), but effects often diminish when formal practice stops or when workers return to high-demand environments (Ong et al., 2024). Current explanations attribute this to skill decay, insufficient dosing, or the need for ongoing practice to maintain benefits. But if mindfulness represents a genuinely acquired skill, we should expect the kind of retention observed for other skills; a gradual decline after practice cessation rather than a rapid regression to baseline (Arthur et al., 1998).

Abstraction habituation offers a more precise explanation. The skill-practice distinction is useful here: a musician retains the skill of playing an instrument even when not performing. Similarly, mindfulness training may successfully cultivate the skill—the capacity for concrete, present-moment processing—and that capacity likely persists as other trained skills do. The problem may not be skill decay as much as environmental pressure. While mindfulness practice cultivates concrete processing, the work environment continuously trains abstraction as the default mode. Eight or more hours daily of abstract work demands versus twenty minutes of mindfulness practice represents an asymmetry heavily favoring abstraction. Effects fade rapidly not because the skill decays but because the environment continuously retrains the default. This explains why ongoing practice is required: not to prevent skill decay, but to counteract the environmental pressure that continuously reinstates abstraction as the automatic mode. The worker retains the capacity for mindful presence but must swim against the current of daily cognitive training to access it.

### 8.4 The stability paradox

Early burnout theory proposed that burnout should be progressive—worsening over time as chronic stress progressively depletes psychological resources (Golembiewski et al., 1986; Maslach, 1982). Yet longitudinal research consistently finds that burnout remains remarkably stable for most workers, with the majority of variance occurring between persons rather than within persons over time (Dunford et al., 2012). Cross-sectional data reveal a mid-career peak, with rates declining partially in late career (Mehta et al., 2019). This pattern—elevated stability with a mid-career peak followed by partial late-career decline—presents a puzzle for resource-based models. If burnout results from progressive resource depletion, why does it plateau rather than continue worsening? If recovery mechanisms exist, why do not accumulated resources, autonomy, and professional mastery in late career restore functioning more substantially?

Abstraction habituation offers a parsimonious explanation for both the stability and the trajectory. Habituation is not progressive depletion but rather a recalibration that reaches equilibrium. Early-career workers retain cognitive flexibility from diverse pre-work experiences—education, unstructured time, less specialized cognitive demands—that maintain concrete processing capacity. The mid-career peak represents maximum habituation: years of daily abstract processing have progressively weakened concrete processing networks, but workers have not yet exited (survivor bias) or transitioned to less abstract roles. The stability observed in longitudinal studies reflects habituation reaching its ceiling; once cognitive systems have recalibrated to abstract processing as the default mode, further exposure produces diminishing additional habituation. Late-career “improvement” in cross-sectional data likely reflects selection effects rather than recovery, as those most habituated have already left.

## 9 Implications

### 9.1 Implications for burnout theory

Abstraction habituation represents a qualitatively distinct cognitive pathway to burnout, complementing rather than replacing existing frameworks. This is not simply another demand to incorporate into JD-R formulations, but a different kind of mechanism; habituation rather than depletion. The type of demand matters, not only the quantity. The JD-R and COR theories retain validity for burnout driven by excessive workload, inadequate support, or resource-demand imbalance. Abstraction habituation adds a mechanism for burnout driven by a specific nature of cognitive demands, when burnout occurs even when resources appear adequate and the workload seems manageable. The models may operate simultaneously: a worker may experience both resource depletion and cognitive habituation, with different intervention implications for each component. The contribution of the present framework lies in explaining phenomena that the resource models struggle to fully address: why recovery fails despite adequate time off, why burnout remains stable despite accumulating resources across careers, and why interventions that work temporarily often fail to produce lasting change.

The model fundamentally reframes mindfulness in the burnout context. Traditional framing positions mindfulness as a personal resource that workers can acquire through training, adding to their capacity to manage demands. The abstraction habituation framing positions mindfulness as a natural cognitive baseline that modern knowledge work environments systematically erode. The intervention logic shifts entirely: rather than adding countermeasures to help workers cope with demanding environments, the priority becomes preventing environments from eroding capacity in the first place. Responsibility shifts from individual skill acquisition to environmental design. This does not diminish the value of mindfulness practice—temporary restoration of concrete processing capacity genuinely helps—but it contextualizes that practice within a larger environmental system.

### 9.2 Implications for intervention

If abstraction habituation operates as proposed, individual-focused mindfulness programs alone cannot address burnout among knowledge workers. Such programs restore concrete processing capacity temporarily, but the environment continues training abstraction (Meaden, 2020; Meaden, 2024b). Programs that ignore work design treat symptoms while the causes continue to operate. The most effective and robust improvement may require combining individual practice with environmental modification.

Environmental redesign could reduce abstraction habituation by targeting each of the three work characteristics identified above. To address *abstraction load*, organizations can introduce concrete components into otherwise abstract work: tangible representations of progress, physical artifacts that ground conceptual projects, embodied activities interspersed with symbolic tasks, and visual dashboards that connect metrics to real-world outcomes^2^. To address *feedback deprivation*, organizations can increase feedback salience through clear success markers, frequent and unambiguous performance signals, and team structures that provide immediate social feedback in lieu of delayed task feedback. To address *closure deprivation*, organizations can design natural completion points into workflows—discrete deliverables that can be genuinely finished, rituals marking task completion, and boundaries on digital accessibility that allow work to be mentally released. Additionally, workspaces incorporating nature exposure could leverage documented benefits of natural environments for concrete processing. The goal is not eliminating abstraction from work—much knowledge work necessarily involves abstract processing—but balancing abstraction with sufficient concrete experience to restore and maintain cognitive flexibility.

Recovery from established abstraction habituation may require deliberate engagement with concrete, sensory-rich activities sufficient for cognitive retraining rather than a focus on relaxation and stress reduction. Brief periods of relaxation are likely insufficient, as cognitive patterns consolidated over years of daily reinforcement require sustained countertraining, not momentary respite. What may be needed is sustained, consistent engagement with activities that actively train concrete processing (e.g., hands-on craftwork, nature-based activities, mindfulness and yoga practices). This also means that non-work time matters: if passive leisure (screens, media consumption) fails to sufficiently engage concrete processing, workers may receive minimal concrete engagement even with abundant free time. Work-life design that ensures sufficient concrete experience may become part of effective burnout prevention.

In support of these propositions, clinical psychology increasingly recognizes the therapeutic value of training concrete processing through select activities. For example, Watkins’ Rumination-Focused Cognitive Behavioral Therapy (Watkins, 2016) employs “concreteness training” which explicitly trains depressed patients away from abstract analytical processing (“why am I feeling this way?”) toward concrete experiential processing (“what exactly am I sensing right now?”), with multiple RCTs demonstrating efficacy for depression treatment (Watkins et al., 2011) and prevention in adolescents and young adults (Topper et al., 2017). In addition, the growing clinical adoption of somatic therapies (Rosendahl et al., 2021), nature-based interventions (Coventry et al., 2021), forest bathing programs (Kotera et al., 2022), and embodied practices like equine-assisted learning (Fuller-Lovins et al., 2023) reflects a societal recognition that concrete, sensory-grounded experience restores something beneficial to well-being that modern life erodes. Abstraction habituation provides a theoretical mechanism explaining why these diverse interventions work: they counteract environmental training toward abstraction by retraining concrete processing capacity, and thereby restore cognitive flexibility.

### 9.3 Implications for research

The model generates specific research directions and testable predictions. First, new measures are needed. For example, an Abstraction Habituation Scale assessing cognitive flexibility between processing modes, distinct from general cognitive flexibility measures. Utilizing such a measure, experience sampling studies could track processing mode throughout the day (i.e., moments of concrete versus abstract engagement) relating patterns to environmental contexts and burnout symptoms. Career trajectory studies could examine cognitive flexibility decline over tenure in more abstraction demanding versus more cognitively balanced occupations (e.g., park ranger, construction manager). Longitudinal designs tracking cognitive mode along with burnout symptoms could test whether habituation mediates the relationship between abstract work demands and burnout outcomes.

The abstraction habituation model of burnout generates falsifiable predictions distinguishing it from alternative explanations. First, cumulative abstract work exposure should predict cognitive inflexibility controlling for workload intensity; a consultant and a tradesperson working equivalent hours should show different flexibility profiles. Second, cognitive inflexibility should mediate the relationship between abstract work and burnout; controlling for inflexibility should reduce the direct effect of abstract work on burnout. Third, burnout in more abstraction-demanding occupations should increase with career tenure before plateauing as habituation reaches its ceiling; whereas more concrete-demanding occupations should show lower peak levels and earlier stabilization due to ongoing environmental recovery opportunities. Fourth, concreteness-training interventions should prove more effective than equal-time passive rest for workers with high habituation, and the difference should be larger for more habituated workers. Fifth, environmental redesign interventions should produce more lasting effects than individual mindfulness training alone, while combinations should outperform either alone. These predictions enable empirical testing and, importantly, distinguish the abstraction habituation model from accounts that treat all demands as qualitatively equivalent or burnout as mostly due to resource depletion.

## 10 Limitations and future directions

### 10.1 Empirical validation

This paper presents a theoretical framework, the direct empirical testing of the abstraction habituation model of burnout awaits future research. The model integrates findings from multiple established literatures (e.g., predictive processing, mindfulness, occupational burnout, environmental psychology) but extends them in ways that require empirical validation.

Operationalizing abstraction habituation for direct measurement might present methodological challenges. While cognitive flexibility can be assessed through established task-switching paradigms and attention measures, the specific abstract-concrete dimension discussed in this paper requires new validated instruments. Self-report measures should be explored but might be limited if individuals lack insight into their own processing modes. Behavioral and physiological measures (e.g., attention allocation, physiological correlates of concrete versus abstract processing) could help to complement self-report, though these approaches require specialized equipment, trained personnel, and controlled laboratory settings. Development of a validated Abstraction Habituation Scale, demonstrating discriminant validity from related constructs like cognitive load, stress, and general fatigue, is a research priority.

### 10.2 Moderators and boundary conditions

The model proposes a universal mechanism—how human cognition responds to environments demanding sustained abstraction—but individual differences and boundary conditions likely moderate its operation. Several factors may influence susceptibility to habituation. Baseline cognitive flexibility likely matters: workers who begin careers with greater capacity for mode-switching may resist habituation longer. The same may be true for individuals high in trait mindfulness. Early-career concrete experience—whether from manual labor, trades, athletics, or other embodied work prior to entering knowledge work—may establish more robust concrete processing networks that resist atrophy.

Several factors may provide protection against habituation, even during knowledge work careers. Manual hobbies (woodworking, gardening, crafts), outdoor activities (hiking, sports, nature immersion), and embodied practices (martial arts, dance, yoga) may provide sufficient concrete processing engagement to counteract workplace training effects. The ratio matters: workers whose non-work hours involve substantial concrete engagement may maintain more cognitive flexibility.

Certain work conditions may prevent or slow habituation. Roles with immediate, tangible feedback, even within knowledge-intensive organizations, may preserve concrete processing capacity. Shorter career tenure in abstract work, by definition, provides less cumulative training. Organizations that build concrete elements into knowledge work (physical prototypes, site visits, hands-on components) may protect workers from abstraction habituation.

Cultural context also requires attention. This framework focuses on knowledge work environments prevalent in industrialized Western economies. Cultures with stronger traditions of embodied practice, closer connection to natural environments, or different work-life boundaries may show different patterns. The EMM’s evidence from non-Western populations (Thong et al., 2024) suggests the abstract-concrete training mechanism operates cross-culturally, but the specific manifestation in occupational burnout requires investigation across diverse contexts.

Future research should systematically identify moderators and boundary conditions rather than assuming uniformity. Understanding who is protected and why may illuminate intervention targets as effectively as understanding the core mechanism itself.

## 11 Conclusion

This paper introduced abstraction habituation as a distinct cognitive pathway to burnout in knowledge workers. The model proposes that sustained abstract work trains minds toward abstraction through neuroplastic adaptation, progressively eroding the concrete processing capacity that supports psychological recovery. Because this represents habituation rather than depletion, the model suggests that recovery requires active retraining, not merely rest.

The framework explains phenomena that resource-based models struggle to address: why recovery fails despite adequate time off, why burnout stabilizes rather than recovering, and why mindfulness intervention effects fade when practice stops. The model generates falsifiable predictions regarding the relationship between abstraction exposure and cognitive flexibility, the mediating role of flexibility in burnout development, and the differential effectiveness of concrete engagement versus passive rest. These predictions enable empirical testing and refinement.

For burnout theory, the model contributes a qualitatively distinct mechanism, cognitive habituation through environmental training, which is intended to complement rather than replace resource frameworks. For intervention, it suggests that lasting improvement may require redesigning work environments to preserve cognitive flexibility, not merely helping individuals cope with environments that erode it. As knowledge work continues to expand globally, understanding how work environments shape the cognitive capacities on which recovery depends becomes both a theoretical priority and a practical necessity.

## Data Availability

The original contributions presented in the study are included in the article/supplementary material, further inquiries can be directed to the corresponding author/s.

## Footnotes

1 For example, hands-on cooking or woodworking would qualify, while strategy-focused gaming or social media scrolling likely would not, despite their absorbing qualities.

2 These features parallel elements of gamification design. However, the framework suggests an important distinction: gamification that connects work to tangible outcomes may support cognitive flexibility, while purely symbolic systems (points, badges, leaderboards disconnected from real-world consequences) may simply compound abstraction [see Koivisto and Hamari (2019), for a review of gamification’s mixed effects].
